# A Generalized Nomenclature Scheme for Graphene Pores, Flakes, and Edges, and an Algorithm for Their Generation and Numbering

**DOI:** 10.3390/nano13162343

**Published:** 2023-08-15

**Authors:** Zacharias G. Fthenakis

**Affiliations:** 1 Istituto Nanoscienze, Consiglio Nazionale delle Ricerche (CNR), 56127 Pisa, Italy; zacharias.fthenakis@nano.cnr.it or fthenak@eie.gr; 2 Theoretical and Physical Chemistry Institute, National Hellenic Research Foundation, 11635 Athens, Greece; 3 National Enterprise for nanoScience and nanoTechnology (NEST), Scuola Normale Superiore, 56127 Pisa, Italy

**Keywords:** nanographenes, graphene flakes, carbon dots, graphene quantum dots, graphene edges, graphene nanoribbons, honeycomb lattice, nomenclature, algorithm, graph theory

## Abstract

In the present study, we generalize our recently proposed nomenclature scheme for porous graphene structures to include graphene flakes and (periodic) edges, i.e., nanographenes and graphene nanoribbons. The proposed nomenclature scheme is a complete scheme that similarly treats all these structures. Beyond this generalization, we study the geometric features of graphene flakes and edges based on ideas from the graph theory, as well as the pore–flake duality. Based on this study, we propose an algorithm for the systematic generation, identification, and numbering of graphene pores, flakes, and edges. The algorithm and the nomenclature scheme can also be used for flakes and edges of similar honeycomb systems.

## 1. Introduction

Although graphene is a semi-metal, graphene flakes (also called nanographenes) and graphene nanoribbons, which are the zero- and one-dimensional counterparts of graphene, are semiconductors, due to quantum confinement. This makes them particularly useful for optoelectronic applications [1] and explains why they have attracted a lot of interest recently [1,2,3,4,5,6,7,8,9,10,11,12,13,14,15,16,17,18,19,20]. In the last decade, several such structures have been synthesized and both top-down and bottom-up methods have been developed for their fabrication [2,4,5,6,7,8,21].

Depending on their sizes, shapes, and edge structures, these structures exhibit a variety of different electronic, optical, and chemical properties [1,10,22]. For instance, graphene nanoribbons with zig-zag termination were found to exhibit localized edge states, while their armchair counterparts do not [23], and for a mixed edge profile, having both zig-zag and armchair sites, non-negligible edge states were found to survive. The different electronic properties of graphene edges provide different reactivity and functionalization [24,25], as well as different reconstruction [26,27], and their optical properties make them potential candidates for optoelectronic, photocatalytic, and other applications [1,3]. Moreover, periodic arrangements of graphene nanostructures with different shapes are potential metamaterial absorbers based on surface plasmon resonance [28,29,30,31].

The infinite number of nanographenes and graphene nanoribbons, due to their different edge structures, and the increasing interest in them, give rise to the necessity for a nomenclature scheme, which would have the ability to accurately distinguish them from each other and provide a particular and accurate name for each such different structure.

So far, attempts for naming nanographenes are restricted to (a) the smaller members of polycrystalline aromatic hydrocarbons and (b) high symmetry flakes. For the former, empirical names have been adopted, for instance, coronene, ovalene, pentacene, pyrene, anthracene, etc. [10]. For the latter, there were attempts to classify them according to the number of C atoms in zig-zag and armchair-terminated hexagonal and/or trigonal flakes [14,16], or (for the hexagonal flakes) to name them after the number of hexagon layers around a central hexagon [15]. However, none of these attempts are able to cover the variety of nanographene structures. On the other hand, there is already a well-established name for two types of graphene edges, the so-called zig-zag and armchair edges [18], which, however, are not the only edges that may appear in nature. An attempt to name edges using the sequence of zig-zag and armchair units along the edge boundary, as described in [23], lacks generality, as it cannot accommodate edges not exclusively formed by zig-zag and/or armchair units. Thus, none of those attempts provide a general scheme to uniquely identify and name flakes and edges. Moreover, some interesting recommendations from the international editorial team of *Carbon*, regarding the nomenclature of graphene-based two-dimensional materials [32], mainly apply to a few-layered or multi-layered graphene flakes or nanosheets. A similar attempt within the European Union GRAPHENE Flagship project [33] suggested a classification based on three raw descriptors, which are the carbon–oxygen (C/O) ratio, the average lateral dimension, and the number of layers. Although, in these studies, it is generally admitted that the nomenclature should be based on morphological descriptors, the proposed descriptors are rather based on raw morphological characteristics, thus, allowing classification, but not allowing a unique description at the atomic level. Therefore, there is a need for a general, accurate, and straightforward nomenclature scheme for graphene flakes and edges, following the IUPAC specifications, which currently do not exist.

Recently, we proposed a nomenclature scheme for porous graphene structures based on the arrangement of two-fold coordinated atoms (2-FCAs) in the pore boundary [34]. In the present work, we generalize that scheme, showing that it may include both graphene flakes and (periodic) edges. Due to the structure of this nomenclature scheme, it allows systematic numbering and recording of both graphene flakes and edges, which, in turn, allows a systematic study of their properties. Moreover, considering that flakes and pores can have the same edge boundary, flakes can be considered as pore duals and vice versa. As we explain, on the other hand, not all pores have flakes as their dual, and not all flakes have pores as their dual. That duality, however, allows the application of the same strategy for naming both flakes and pores, even in cases where a dual structure does not exist. This is what we show and use in the present study. In addition, the same strategy can be used to name infinite graphene edges (i.e., edges extended to infinity, not forming a closed path), which might or might not be periodic, taking into account that edges do not have a closed boundary.

It is worth noting that the proposed nomenclature scheme and the generation and numbering algorithm apply to graphene flakes and edges, which are composed of hexagonal rings, including either three-fold coordinated atoms (3-FCAs) or two-fold coordinated atoms (2-FCAs) only. Flakes or edges, including (i) atoms bonded with only one atom, (ii) parts of the flake (or the edge) bonded with the rest of the flake (or the edge) system with only one bond, (iii) helical-like flakes [9], or (iv) flakes containing non-hexagonal rings, are not taken into account in the present study. These cases include extra complexity and they should be studied separately. The nomenclature scheme proposed here offers just the basis on which nomenclature schemes for more complex cases can be built. However, an expansion of the proposed nomenclature scheme for flakes and edges, to cover edge functionalization and/or edge reconstruction, is possible, in accordance with the expansion proposed in the case of graphene pores [34].

## 2. Materials and Methods

### 2.1. From Graphene Pores to Graphene Flakes and Edges

In the current section, we show the connection between flakes and edges with pores, which has been studied previously [34]. Considering that, in terms of graph theory, a graphene pore is a non-crossed closed path composed of *edges* (bonds) and *vertices* (atoms), we showed that each such closed path (i.e., circuit) can be uniquely defined by the sequence of path lengths $l_{i}$ along the pore boundary connecting the 2-FCAs [34].

The term “*non crossed*” means that if we move along that path, each vertex and edge is visited only once. The term “*closed path*” or “*circuit*” means that the path begins and ends at the same vertex. In terms of graph theory, these properties describe both an *Eulerian* and *Hamiltonian circuit*. The term “*path length*” is also used from the point of view of graph theory, i.e., it is the number of edges along the path. For convenience, we use the term “*2-2 path*” for the path along the pore boundary connecting two adjacent 2-FCAs, and the term “*2-2 hexagon*” for the hexagon of the vacuum space associated with the 2-2 path, which contains the 2-2 path, i.e., the edges of the 2-2 path are edges of the associated 2-2 hexagon. As we showed in our recent work [34], the length $l_{i}$ of a 2-2 path *i* is an integer number between 1 and 4 ($1\leq l_{i}\leq 4$), and the sequence $l_{1}l_{2}\ldots l_{n_{2}}$ of the 2-2 path lengths $l_{i}$ uniquely determines a pore. However, there are many different sequences corresponding to the same pore, depending on the starting point and the direction one uses to travel along the pore boundary. For a one-to-one correspondence between pores and sequences, we select the “*minimum image*” of all possible sequences that one can find, starting from a different vertex of the circuit, and moving along it clockwise or counterclockwise. Considering that the different sequences $l_{1}l_{2}\ldots l_{n_{2}}$ corresponding to the same pore are $n_{2}$-digit numbers composed of the $l_{i}$ digits ($1\leq l_{i}\leq 4$), the “minimum image” is the sequence representing the minimum of those numbers. According to the nomenclature scheme we proposed, a pore is named after the unique sequence of 2-2 path lengths $l_{i}$ provided by the “minimum image”. That scheme provides an accurate, simple, and unique way to name pores. A pore with the minimum image sequence $l_{1}l_{2}\ldots l_{n_{2}}$ was proposed to be named as “*the $l_{1}l_{2}\ldots l_{n_{2}}$ pore*”. The same nomenclature scheme is used here for naming graphene flakes and edges.

In Ref. [34], for pores, we introduced the term “*edge vectors*” $e_{k}$. An edge vector $e_{k}$ is a vector attributed to the edge *k* of the pore circuit, where *k* serially numbers all edges (or vertices) of the pore boundary. The direction of $e_{k}$ is the direction one has, moving along the pore boundary counterclockwise, so that, if $e_{k}$ is directed from left to right, bottom to top, right to left, or top to bottom, the vacuum pore area is above, on the left, below, and on the right of the edge vector, respectively. For those edge vectors, we show that each vector $e_{k}$ of the pore boundary can be derived from the previous $e_{k-1}$ vector. In particular, if the origin of $e_{k}$ (or equivalently the head of $e_{k-1}$) coincides with the position of a 3-FCA, then $e_{k}$ can be derived by the rotation of $e_{k-1}$ counterclockwise by +π/3. On the other hand, if it coincides with the position of a 2-FCA, then $e_{k}$ can be derived by the rotation of $e_{k-1}$ counterclockwise by −π/3. Adopting the alternative notation $e_{i,j}$ for the edge vectors $e_{k}$, where *i* denotes the 2-2 path and *j* the edge vector belonging to the *i* 2-2 path, and considering that R(ϕ) is the 2×2 rotation matrix for rotations in-plane by ϕ, we have $e_{i,j+1}=R_{+}e_{i,j}$, for 1≤j≤4 and $1\leq i\leq n_{2}$, and $e_{i+1,1}=R_{-}e_{i,l_{i}}$, for $1\leq i\leq n_{2}$. In those relations, $R_{\pm }=R\left(\pm \pi /3\right)$. Recalling that for in-plane rotations R(ϕ)R(θ)=R(θ)R(ϕ)=R(ϕ+θ) and taking into account that for a closed path, $e_{n_{2}+1,1}=e_{1,1}$, we find $R_{-}^{n_{2}}R_{+}^{n_{3}}=R_{-}^{n_{2}}R_{+}^{L-n_{2}}=I$, where *I* is the 2×2 unit matrix and $L=l_{1}+l_{2}+\ldots +l_{n_{2}}$ is the total length of the pore circuit. Thus, $-n_{2}\pi /3+\left(L-n_{2}\right)\pi /3=2k_{0}\pi$, $k_{0}\in Z$ or

(1) $$L=2n_{2}+6k_{0},\;k_{0}\in Z.$$

In Ref. [34], we show that for pores, if $e_{n_{2}+1,1}=e_{1,1}$, then

(2) $$L-2n_{2}=6.$$

Equation (1) leads to the same result if $k_{0}=1$. However, Equation (1) shows that there are also many other solutions, depending on $k_{0}$, for which $e_{n_{2}+1,1}=e_{1,1}$. The question, therefore, which rises, is what do the other cases for $k_{0}\neq 1$ represent? These cases will be examined next.

**Figure 1:**
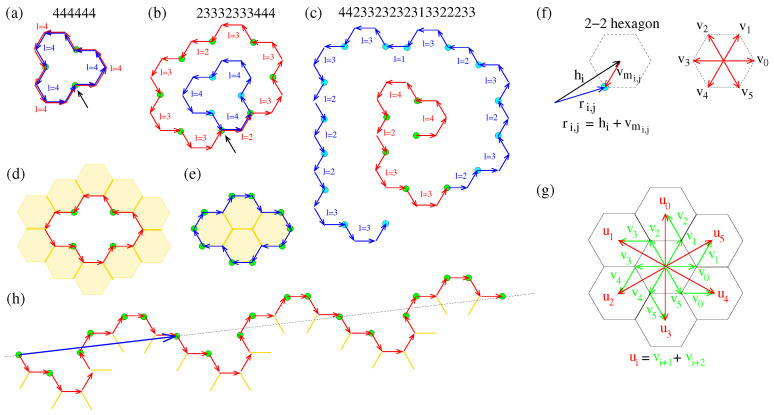
(**a**–**c**) Circuits and a path with $L=2n_{2}+12$. (**a**) The 444444 circuit composed of two 444 pore circuits overlapping each other. (**b**) The 233233444 circuit composed of the 233233 and 444 circuits crossing each other. (**c**) The non-closed 4423323232313322233 path forming a spiral. Different colors of edge vectors and 2-FCAs indicate the first and second circle (i.e., the path for which $L=2n_{2}+6$). The black arrow in (**a**,**b**) indicates the starting vertex. (**d**,**e**) A pore and its dual flake, respectively. Shaded hexagons and dark yellow colored sticks represent the graphene bulk area and bonds between the three-fold coordinated atoms. Red and blue colored arrows represent edge vectors in the pore and flake boundaries, respectively. Red-colored edge vectors have opposite directions compared to their blue counterparts. (**h**) Periodically arranged edge vectors with $L=2n_{2}$ forming an edge. The blue arrow represents the period.

As we have shown in Ref. [34], $e_{i,j}$ can be expressed through a rotation of $e_{1,1}$. In particular, we showed that

(3) $$e_{i,j}=R_{-}^{i-1}R_{+}^{k-i}e_{1,1},$$

where

(4) $$k=\left(1-\delta_{i,1}\right)\sum_{i^{\prime }=1}^{i-1}l_{i^{\prime }}+j,$$

where $\delta_{i,j}$ is the Kronecker δ. Thus, if $e_{i,j}=R\left(\phi_{i,j}\right)e_{1,1}$, then $\phi_{i,j}=\left(k-i\right)\pi /3-\left(i-1\right)\left(-\pi /3\right)$, or

(5) $$\phi_{i,j}=\left(k-2i+1\right)\pi /3,$$

and, consequently, $\phi_{n_{2}+1,1}=\left(L+1-2\left(n_{2}+1\right)+1\right)\pi /3=\left(L-2n_{2}\right)\pi /3$. Thus, using Equation (1), we find $\phi_{n_{2}+1,1}=6k_{0}\pi /3=2k_{0}\pi$, which for $k_{0}=1$ represents the angle of the full (2π) rotation of $e_{1,1}$, which has already been examined in Ref. [34]. Therefore, for $k_{0}>1$, the rotation angle $\phi_{n_{2}+1,1}$ of $e_{1,1}$ represents more than one full rotation. In that case, there are three options: (i) The pore circuit closes earlier, having the first full rotation for $i=i_{0}<n_{2}+1$, i.e., $\phi_{i_{0}+1,1}=2\pi$, (ii) the pore circuit crosses itself, or/and (iii) it forms a spiral. These cases obviously do not correspond to Eulerian and Hamiltonian paths, i.e., moving along those paths, either we visit the same vertex (or vertices) and edge (or edges) more than once, and/or the path does not close. A simple example of a circuit that closes earlier is a circuit that is repeated after its end, e.g., the 444444 path. The 444 path represents the smallest pore, which is formed if a single atom is removed from the graphene structure. For the 444 pore, L=12 and $n_{2}=3$, satisfying Equation (2). The 444444 path is also a circuit, with L=24 and $n_{2}=6$, satisfying Equation (1) for $k_{0}=2$. Moving along that path, we visit its vertices and edges twice, as shown in Figure 1a and, consequently, the path does not represent a pore. An example of a circuit that crosses itself is the circuit 23332333444, which is presented in Figure 1b. Both 444 and 23332333 paths represent pores and they are shown with different colors in Figure 1b. The 444 has already been mentioned above, and for the 23332333 path, L=22 and $n_{2}=8$, also satisfying Equation (2). The 23332333444 path, with L=34 and $n_{2}=11$, satisfies Equation (1) for $k_{0}=2$. Moving along this path, we visit three vertices and two edges twice. An example of a spiral path, (which is not a circuit), is the 442242222422222422222242222224 path, which is shown in Figure 1c. This spiral path satisfies Equation (1) for $k_{0}>1$, while its vertices and edges are not visited more than once, as we travel along it.

In conclusion, cases for $k_{0}>1$ represent either spiral or crossed paths, with no interest in the present work since they do not represent pores.

#### 2.1.1. Graphene Flakes

The case for $k_{0}=-1$ corresponds to a rotation angle $\phi_{n2+1,1}=-2\pi$, which corresponds to a clockwise rotation. Following the same convention as the one used for pores, regarding the relative position of the vacuum space with respect to the direction of the edge vectors, it is easy to understand that a closed and non-crossed path with $\phi_{n2+1,1}=-2\pi$ forms a flake, as can be seen in Figure 1e. For comparison, Figure 1d shows its counterpart pore structure. In accordance with paths or circuits corresponding to rotation angles $\phi_{n2+1,1}=2k_{0}\pi$, with $k_{0}>1$, the paths or circuits corresponding to rotation angles $\phi_{n2+1,1}=2k_{0}\pi$ with $k_{0}<-1$ are similar but rotated clockwise. In conclusion, no interesting circuits or paths can be obtained for $k_{0}<-1$, while the case for $k_{0}=-1$, which yields

(6) $$L=2n_{2}-6,$$

corresponds to graphene flakes.

As in pores, where Equation (2) is a necessary but not sufficient condition for a closed path [34], Equation (6) is a necessary, but not sufficient condition for a closed path in the flakes. In accordance with our study for pores [34], the position vectors of the vertices (atoms) at the flake boundary are

(7) $$r_{i,j}=h_{i}+v_{m_{i,j}},$$

where

(8) $$v_{m}=a_{0}\left(\cos\frac{m\pi }{3},\sin\frac{m\pi }{3}\right),\;m=0,1,2,3,4,5,$$

and $h_{i}$ is the position vector of the center of the 2-2 hexagon *i*, as shown in Figure 1f. The indices *i* and *j* are the same as those used in the notation of the edge vectors $e_{i,j}$, and

(9) $$e_{i,j}=v_{mod(m_{i,j}+2,6)}.$$

$m_{i,j}$ are integer numbers between 0 and 5, with

(10) $$m_{i,j}=mod\left(m_{1,1}+k-2i+7,6\right),$$

where *k* is given in Equation (4). Moreover,

(11) $$h_{i+1}=h_{1}+\sum_{j=1}^{i}u_{m_{j,l_{j}}},$$

where

(12) $$u_{m}=\sqrt{3}a_{0}\left(-\sin\frac{m\pi }{3},\cos\frac{m\pi }{3}\right),\;m=0,1,2,3,4,5,$$

as shown in Figure 1g, (see Ref. [34] for more details). For a closed path, $h_{n_{2}+1}=h_{1}$, i.e.,

(13) $$\sum_{i=1}^{n_{2}}u_{m_{i,l_{i}}}=0,$$

where according to Equations (10) and (4),

(14) $$m_{i,l_{i}}=mod\left(m_{i-1,l_{i-1}}+l_{i}+4,6\right).$$

Equations (2) and (13) are the necessary and sufficient conditions for a close path. However, they still are not enough to determine a flake boundary, since they do not ensure that the path is not a crossed path. As we showed for pores [34], a non-crossed path is the one for which (i) two 2-2 hexagons do not have common edges belonging to the pore boundary and (ii) traveling along the pore boundary, the corresponding 2-2 hexagons are not visited more than once unless some of them are visited twice. For those visited twice, the lengths of the associated 2-2 paths must be 1 and their sole edge vectors must have opposite directions. For the same reason, these conditions should also be satisfied for flakes.

#### 2.1.2. Graphene Edges

For $k_{0}=0$, Equation (1) yields $L/n_{2}=2$. In terms of the rotating angle $\phi_{i,j}$, one can see that $\phi_{n_{2}+1,1}=0$. Assuming that a path with $\phi_{n_{2}+1,1}=0$ is not a crossed path, the edge vectors of that path will be rotated negatively and positively, so that at the end, the overall rotation angle will be zero. Even if for some specific *i* and *j* indices of $e_{i,j}$, $\phi_{i,j}\geq 2\pi$, or $\phi_{i,j}\leq -2\pi$, these rotations will be followed by opposite rotations, which at the end will lead to a zero overall rotation for the final edge vector $e_{n_{2}+1,1}$. This means that that path will not be able to close and, therefore, it does not represent a pore or a flake. Assuming that the $l_{1}l_{2}\ldots l_{n_{2}}$ sequence is repeated, as can be assumed in a pore or a flake circuit, the starting edge vector $e_{n_{2}+1,1}$ of the first repetition will be the same as the starting edge vector $e_{1,1}$; consequently, the whole repeated path will be arranged parallel to the initial $l_{1}l_{2}\ldots l_{n_{2}}$ path. This will also happen for all repetitions that follow, and the overall path will form a periodic edge, which divides the plane into the vacuum and a semi-infinite graphene structure (see Figure 1h). In conclusion, the case $k_{0}=0$ of Equation (1) can be considered as corresponding to an infinite periodic graphene edge, which is obviously characterized by the $l_{1}l_{2}\ldots l_{n_{2}}$ sequence, and can be named after it. For a one-to-one correspondence between edge names and $l_{1}l_{2}\ldots l_{n_{2}}$ sequences, the minimum image could be selected for the smallest period. In that case, $n_{2}$ is the number of 2-FCAs (or 2-2 paths) in that smallest periodic arrangement of atoms at the boundary, e.g., the edge 131313… will be named “the 13 edge”, with $n_{2}=2$.

As in pores and flakes, a boundary path representing an edge should not be a crossed path. The conditions for a non-crossed path in the edges are the same as those in pores and flakes.

It is worth noting that in the case of a path $l_{1}l_{2}\ldots l_{n_{2}}$ corresponding to a graphene edge, the average length of 2-2 paths $L/n_{2}$ is $L/n_{2}=2$, while for pores and flakes, $L/n_{2}>2$ and $L/n_{2}<2$, respectively.

### 2.2. Flakes and Flake–Pore Duality

Obviously, a circuit representing a pore boundary can be either considered the boundary of a pore, or the boundary of a flake, depending on the side where the vacancies are. A flake boundary can be considered (in several cases) the dual of a pore boundary, and vice versa, although, as we have already mentioned in the introduction and will explain below, not all pores have a flake as a dual, and not all flakes have a pore as a dual.

If the dual exists, then for the transformation of a pore to its dual flake, and the transformation of a flake to its dual pore, we consider the following:

- The dual of the dual of a pore (flake) is the pore (flake) itself. As a consequence, the dual is unique, i.e., there is a one-to-one correspondence between a pore and its dual flake and between a flake and its dual pore. If this is not true, we consider that the dual of a pore or flake does not exist.
- The pore (or flake) boundary is the same as that of its dual. The difference is that the edge vectors of the pore (flake) boundary have opposite directions compared to those of its dual flake (pore). This convention for the direction of edge vectors is used so that we have the same determination of the vacuum area and graphene bulk area, regarding their relative position, with respect to the direction of the edge vectors (see above).
- Excluding the atoms of the boundary, all other atoms are transformed into vacancies and vacancies are transformed into atoms.

The above can be seen in Figure 1d,e, which schematically shows a pore and its dual flake, respectively. As a consequence of the above, the boundary of the pore (flake) dual is again formed by a sequence of 2-2 paths, which are different from the 2-2 paths of the pore (flake) boundary, but they both have the same number of edges and vertices and, consequently, the same length *L*. Moreover, 2-FCAs of a pore (flake) are transformed into 3-FCAs in its dual flake (pore) and vice versa. Thus, $n_{2p}=n_{3f}$ and $n_{3p}=n_{2f}$, where $n_{2p}$ and $n_{2f}$ are the numbers of 2-FCAs in the pore and its dual flake, respectively, and $n_{3p}$ and $n_{3f}$ are the numbers of 3-FCAs in the pore and its dual flake, respectively.

Considering that $n_{3p}=L-n_{2p}$, Equation (2) yields $n_{3p}-n_{2p}=6$. Thus, using the above relations, $n_{3p}=n_{2f}$ and $n_{2p}=n_{3f}$, Equation (2) turns into $n_{2f}-n_{3f}=6$, or $n_{2f}-\left(L-n_{2f}\right)=6$, or $L-2n_{2f}=-6$, which is exactly the same as Equation (1), for $k_{0}=-1$ and, consequently, consistent with the discussion of Section 2.1.1, for $k_{0}=-1$. Therefore, it is evident that if a pore boundary has $n_{2}$ 2-2 paths, its dual has $L-n_{2}$ 2-2 paths, and vice versa.

**Figure 2:**
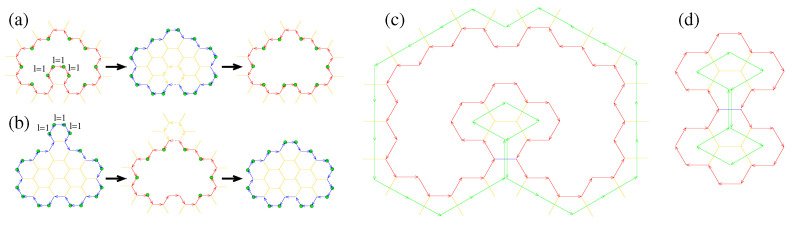
Cases of pores and flakes, which do not have a dual. Yellow sticks represent bonds between 3-FCAs. (**a**) The transformation of a pore to a flake and then the transformation of that flake to a pore. (**b**) The transformation of a flake to a pore and then the transformation of that pore to a flake. Excluding atoms in the pore or flake boundary, which remain atoms, all other atoms are transformed into vacancies and vice versa. The final pore of (**a**) and flake of (**b**) is not the same as the initially transformed one. The blue and red arrows in (**a**,**b**) represent edge vectors at the flake and pore boundaries, respectively. A pore (**c**) and a flake (**d**) with two 3-FCAs, which are not adjacent along the pore or flake boundary, and are bonded with each other. The bond is shown in blue. The circuit formed by connecting the centers of hexagons adjacent to the edges of the pore or flake boundary (green arrows) is not the Eulerian circuit when the dual does not exist.

In Ref. [34], we showed that the maximum number of adjacent 2-2 paths with length $l_{i}=1$ in a pore boundary is three, as shown schematically in Figure 2a. It is worth noting that, excluding the case of the hexagonal 111111 flake, the restriction of the maximum three adjacent 2-2 paths with length $l_{i}=1$ is also valid for flakes, for the same reason as in pores (see Ref. [34]). Moreover, in the same work, we showed that a 2-2 hexagon of a pore cannot be visited more than once as we travel along the path of the pore boundary unless there are only two 2-2 paths corresponding to the same 2-2 hexagon with lengths l=1, and their sole edge vectors having opposite directions. Such a case can be seen in the pore shown schematically in Figure 2c. This restriction also applies to flakes for the same reason it applies to graphene pores.

Let us now examine cases of flakes that do not have a pore as a dual and pores that do not have a flake as a dual. Pores or flakes that include three adjacent 2-2 paths in their boundaries with length $l_{i}=1$ do not have a dual, as shown in Figure 2a,b. In such pores, those three 2-2 paths are edges of the same hexagon, which is part of the graphene bulk. In the transformation of such a pore to its dual flake, the hexagon remains part of the flake. Thus, the derived flake has a different boundary compared to that of the pore. Therefore, the derived flake does not have a one-to-one correspondence with the transformed pore, as Figure 2a shows; consequently, such a pore does not have a dual. For the same reason, a flake with more than two adjacent 2-2 paths with length $l_{i}=1$ does not have a dual pore, as Figure 2b shows.

The above cases are not the only cases for which a pore or a flake does not have a dual. In fact, those cases can be considered as special cases of a more general one, where two 3-FCAs of the pore (or flake) boundary, which are not adjacent along the pore (or flake) path, are bonded with each other. The reason is that the transformation from the pore to the flake (or from the flake to the pore) is expected to be accompanied by the conversion of 3-FCAs to 2-FCAs. However, two such 3-FCAs of the pore or flake boundary will remain 3-FCAs in the transformation from the pore to the flake or from the flake to the pore since their bond will not be lost in the transformation. Thus, those atoms will not be converted to 2-FCAs, and the converted structure will have a different boundary compared to the initial one, not allowing the transformed one to be considered as the dual of the initial one. This can be clearly seen in the examples shown schematically in Figure 2c,d for a pore and a flake, respectively. From a different point of view, a flake or a pore does not have a dual if the closed path connecting the centers of the hexagons, which are adjacent to the pore boundary and are part of the flake or the graphene bulk (for pores), is not the Eulerian circuit. This means that if traveling along that path we visit the same edge more than once, (as shown in Figure 2c,d), then the dual does not exist.

If the dual of a pore (or a flake) exists, the conversion of the pore (or the flake) to its dual implies the replacement of 2-FCA vertices and 2-2 paths of its boundary to other 2-2 paths, according to the following scheme:

(i) The central edge vector $e_{i,2}$ of a 2-2 path *i* with $l_{i}=3$ is replaced by a 2-2 path with length l=1;
(ii) The central edge vectors $e_{i,2}$ and $e_{i,3}$ of a 2-2 path *i* with $l_{i}=4$ are replaced by two adjacent 2-2 paths with length l=1;
(iii) The $e_{i,l_{i}}$ and $e_{i+1,1}$ edge vectors of the 2-2 paths *i* and i+1 with lengths $l_{i}\geq 2$ and $l_{i+1}\geq 2$ are replaced by a 2-2 path with length l=2;
(iv) Two adjacent 2-2 paths *i* and i+1 with lengths $l_{i}=l_{i+1}=1$ appearing between two other 2-2 paths with lengths $l_{i-1}>1$ and $l_{i+2}>1$, and their adjacent edges, $e_{i-1,l_{i-1}}$ and $e_{i+2,1}$, are replaced by a 2-2 path with length l=4;
(v) A 2-2 path *i* with length $l_{i}=1$ appearing between two other 2-2 paths with lengths $l_{i-1}>1$ and $l_{i+1}>1$, and their adjacent edges $e_{i-1,l_{i-1}}$ and $e_{i+1,1}$ are replaced by a 2-2 path with length l=3.

**Figure 3:**
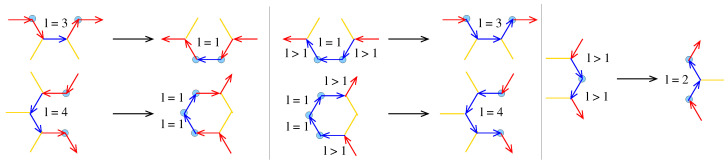
The transformation of 2-2 paths of a pore or flake to their dual. The red and blue arrows represent edge vectors at the pore or flake boundary. Blue-colored edge vectors are those that are transformed. The 2-FCAs are shown with light blue spheres. The dark yellow sticks represent bonds between 3-FCAs.

For example, the dual of the 444 pore is the 112112112 flake. The transformations are shown schematically in Figure 3.

Considering that in a flake circuit with a total length *L*, there are $n^{\left(1\right)}$, $n^{\left(2\right)}$, $n^{\left(3\right)}$, and $n^{\left(4\right)}$ 2-2 paths with lengths 1, 2, 3, and 4, respectively, the total length *L* of the circuit is $L=1\cdot n^{\left(1\right)}+2\cdot n^{\left(2\right)}+3\cdot n^{\left(3\right)}+4\cdot n^{\left(4\right)}$, and the total number of 2-2 paths, $n_{2}=n^{\left(1\right)}+n^{\left(2\right)}+n^{\left(3\right)}+n^{\left(4\right)}$. Recalling that for flakes $L=2n_{2}-6$ and combining all these equations, we find $n^{\left(1\right)}+2n^{\left(2\right)}+3n^{\left(3\right)}+4n^{\left(4\right)}=2\left(n^{\left(1\right)}+n^{\left(2\right)}+n^{\left(3\right)}+n^{\left(4\right)}\right)-6$ or

(15) $$n^{\left(3\right)}+2n^{\left(4\right)}=n^{\left(1\right)}-6.$$

The quantities $n^{\left(3\right)}$ and $n^{\left(4\right)}$ are positive numbers or zero. Thus, the above equation yields $n^{\left(1\right)}-6\geq 0$ or $n^{\left(1\right)}\geq 6$, which means that a flake circuit does not exist, not including at least six 2-2 paths with length l=1.

An interesting question that needs to be answered has to do with the number *N* of atoms constituting a flake. Using the same strategy as the one used to find the number of vacancies in a pore (see Ref. [34]), we can consider that if the number of hexagons constituting a flake is $n_{h}$, then those hexagons have $6n_{h}$ vertices in total. The vertices corresponding to 2-FCAs in the flake boundary are not shared between those hexagons, while the 3-FCAs in the flake boundary are shared by two, and all other 3-FCAs are shared by three. Assuming that the number of all other 3-FCAs is $n_{v}$, then $6n_{h}=n_{2}+2n_{3}+3n_{v}$ and $N=n_{2}+n_{3}+n_{v}$. This means that $6n_{h}=3N-2n_{2}-n_{3}$. Recalling that $n_{2}+n_{3}=L$ and $L=2n_{2}-6$, the last equation yields $6n_{h}=3N-L-n_{2}=3N-2n_{2}+6-n2=3N-3n_{2}+6$, or

(16) $$N=2n_{h}+n_{2}-2.$$

A method used to count the hexagons of a flake is very similar to the method used to count the hexagons in the pore vacuum area, which has been described in Ref. [34]. This method is presented below.

**Figure 4:**
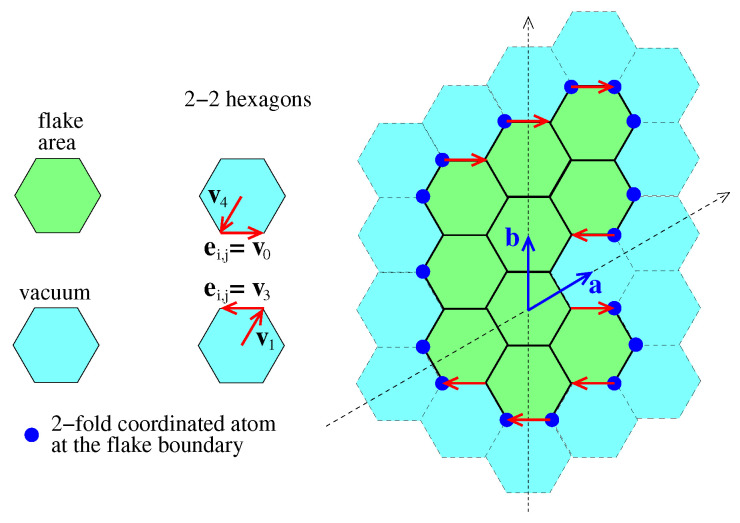
A flake example for the calculation of flake atoms.

Let us assume that the $h_{i}$ vectors determining the 2-2 hexagons are $h_{i}=\lambda_{a,i}a+\lambda_{b,i}b={\left(\lambda_{a,i},\lambda_{b,i}\right)}_{h}$, and $\lambda_{a,i}$ between $a_{min}$ and $a_{max}$, (i.e., $a_{min}\leq \lambda_{a,i}\leq a_{max}$), where $a=\sqrt{3}a_{0}\left(\sqrt{3}/2,1/2\right)$ and $b=\sqrt{3}a_{0}\left(0,1\right)$ are the lattice vectors in the Cartesian coordinates of the hexagonal graphene lattice, as shown schematically in Figure 4, where $a_{0}=1.42$ Å is the bond length in graphene. The subscript *h* of the above notation is used to distinguish the coordinates expressed in the base of a and b from the corresponding Cartesian coordinates. Let us further consider that $h^{\prime }={\left(\lambda_{a}^{\prime },\lambda_{b}^{\prime }\right)}_{h}$ is a vector pointing to the center of a hexagon belonging to the flake. As one can see in Figure 4, $a_{min}+1\leq \lambda_{a}^{\prime }\leq a_{max}-1$. In the same figure, one can see that there are 2-2 paths associated with specific 2-2 hexagons, which include the edge vectors $e_{i,j}=v_{0}$ and $e_{i^{\prime },j^{\prime }}=v_{3}$ depicted by red colored arrows pointing to the right and to the left, respectively. Therefore, it is evident that the hexagons belonging to the flake are those between those 2-2 hexagons, which have as the upper bound the 2-2 path, which includes the $e_{i,j}=v_{0}$ vector, and the lower bound, the one including the $e_{i^{\prime },j^{\prime }}=v_{3}$ vector. Noting that the position vectors of the vertices at the origin of the edge vectors $e_{i,j}=v_{0}$ and $e_{i^{\prime },j^{\prime }}=v_{3}$ are $r_{i,j}=h_{i}+v_{4}$ and $r_{i^{\prime },j^{\prime }}=h_{i^{\prime }}+v_{1}$, respectively, (see Equation (9) and Figure 4), the hexagons belonging to the flake, which are between $h_{i}$ and $h_{i^{\prime }}$ can be equivalently determined by the $m_{i,j}$ values of their associated 2-2 paths. Thus, if (i) for $h_{i}=\left(\lambda_{a}^{\prime },\lambda_{b1}\right),\;\exists \;j\in \left[1,l_{i}\right]\;:\;m_{i,j}=4$, (ii) for $h_{i^{\prime }}=\left(\lambda_{a}^{\prime },\lambda_{b2}\right),\;\exists \;j^{\prime }\in \left[1,l_{i^{\prime }}\right]\;:\;m_{i^{\prime },j^{\prime }}=1$, and (iii) $\lambda_{b1}>\lambda_{b2}$, then there are $\lambda_{b1}-\lambda_{b2}-1$ hexagons belonging to the flake between the 2-2 hexagons determined by $h_{i}$ and $h_{i^{\prime }}$. Those hexagons belonging to the flake can be determined by the vectors $\left(\lambda_{a}^{\prime },\lambda_{b}^{\prime }\right)$, with $\lambda_{b2}+1\leq \lambda_{b}^{\prime }\leq \lambda_{b1}-1$. This can also be used to find the positions of the flake atoms. Summing the numbers of these hexagons for each different $\lambda_{a}^{\prime }$ value in the range $a_{min}+1\leq \lambda_{a}^{\prime }\leq a_{max}-1$, we can find $n_{h}$, and then using Equation (16), we can find *N*.

### 2.3. Edges and Periodic Arranged $l_{1}l_{2}\ldots l_{n_{2}}$ Paths

A graphene edge can be considered a non-crossed, non-closed, and theoretically infinite path, dividing the honeycomb lattice into two discrete areas. One of them only has vacancies and the other one is the remaining graphene structure. Practically, however, a graphene edge is part of the boundary of a typically large graphene flake and not the boundary of a semi-infinite plane. Therefore, in terms of graph theory, a graphene edge is both an Eulerian and Hamiltonian path, which, contrary to the boundaries of flakes and pores, is a path and not a circuit. Such a path should not form a spiral because a spiral might correspond to a non-crossed and non-closed infinite path, but it does not divide the honeycomb lattice plane into the two discrete areas described above.

From the point of view of an infinite path, the graphene edge can be considered periodic. Even if it is not periodic, it can be considered periodic with infinite periodicity. Let us assume that the smallest possible period of a specific edge is represented by the sequence $l_{1}l_{2}\ldots l_{n_{2}}$ of 2-2 paths with length $L=l_{1}+l_{2}+\ldots l_{n_{2}}$, where $n_{2}$ is the number of 2-FCAs in the period. It is worth noting that the length *L* is the period of edge lengths at the boundary. This means that after the sequence $l_{1}l_{2}\ldots l_{n_{2}}$, another such sequence will follow, and the edge will be a repeated sequence of 2-2 paths of the form $\left(l_{1}l_{2}\ldots l_{n_{2}}\right)\left(l_{1}l_{2}\ldots l_{n_{2}}\right)\left(l_{1}l_{2}\ldots l_{n_{2}}\right)\ldots$. Obviously, there is no reason for Equation (3) not to be valid; therefore, the angle $\phi_{i,j}$ between the edge vector $e_{i,j}$ at the graphene edge boundary and the $e_{1,1}$ (first edge vector of the period) is again given by Equation (5). As already reported in Section 2.1, for an infinite (not closed) path, the angle $\phi_{l_{n_{2}}+1,1}$ of the first edge vector of the second period should be $\phi_{l_{n_{2}}+1,1}=0$. If it is not zero, then at each period, the angle will increase (or decrease) by a multiple of π/3, and finally, the path will either cross with itself or will close, thus not forming an infinite periodic edge. For instance, if $\phi_{l_{n_{2}}+1,1}=\pi /3$, then $\phi_{2l_{n_{2}}+1,1}=2\pi /3$, $\phi_{3l_{n_{2}}+1,1}=3\pi /3$, $\phi_{4l_{n_{2}}+1,1}=4\pi /3$, $\phi_{5l_{n_{2}}+1,1}=5\pi /3$, and $\phi_{6l_{n_{2}}+1,1}=2\pi$, and the considered periodic edge will form a pore unless it crosses with itself during the repetitions, which again does not form an edge. Negative multiples of π/3 for $\phi_{l_{n_{2}}+1,1}$ produce flakes and positive multiples produce pores. It is, therefore, clear that for an infinite periodic edge, $\phi_{l_{n_{2}},1}=\phi_{1,1}=0$, corresponding to $k_{0}=0$ in Equation (1), or

(17) $$L/n_{2}=2,$$

which, as already explained, is the average path length.

A question that rises is whether or not there are specific combinations of path lengths for which $L/n_{2}=2$. Assuming that the periodic path is composed by $n^{\left(1\right)}$, $n^{\left(2\right)}$, $n^{\left(3\right)}$, and $n^{\left(4\right)}$ 2-2 paths with lengths 1, 2, 3, and 4, respectively, the last equation yields

(18) $$n^{\left(3\right)}+2n^{\left(4\right)}=n^{\left(1\right)}.$$

The independent integer solutions of this equation are

(i) $n^{\left(1\right)}=0$, $n^{\left(2\right)}=1$, $n^{\left(3\right)}=0$ and $n^{\left(4\right)}=0$;
(ii) $n^{\left(1\right)}=1$, $n^{\left(2\right)}=0$, $n^{\left(3\right)}=1$ and $n^{\left(4\right)}=0$; or
(iii) $n^{\left(1\right)}=2$, $n^{\left(2\right)}=0$, $n^{\left(3\right)}=0$ and $n^{\left(4\right)}=1$.

This means that any combinations of the sets (i) A = {2}, (ii) B = {1, 3} and/or (iii) C = {1, 1, 4} of 2-2 path lengths provide an infinitely periodic path, unless that path crosses itself, and any infinitely periodic path is composed of the combinations of the above sets of 2-2 paths. This does not mean that those sets of 2-2 paths should necessarily follow one another in the periodic $l_{1}l_{2}\ldots l_{n_{2}}$ sequence. The members of those sets may mix with each other, but the sequence $l_{1}l_{2}\ldots l_{n2}$ should be composed of a certain number of those sets. Thus, if $\lambda_{2}$, $\lambda_{13}$, and $\lambda_{114}$ are the numbers of A, B, and C sets in the periodic $l_{1}l_{2}\ldots l_{n_{2}}$ sequence, respectively, then $n^{\left(1\right)}=2\lambda_{114}+\lambda_{13}$, $n^{\left(2\right)}=\lambda_{2}$, $n^{\left(3\right)}=\lambda_{13}$, and $n^{\left(4\right)}=\lambda_{114}$.

It is worth noting that the …2222… edge is the well-known zig-zag edge, while the …131313… is also the well-known armchair edge. Moreover, it is worth noting that when adding any combination of the above sets of 2-2 paths in a pore or flake circuit, Equation (2) (for pores) and Equation (6) (for flakes) are still satisfied. On the other hand, this does not mean that by adding such a combination in the pore or flake boundary circuit, the derived path is still a circuit.

The last task for periodic edges is the construction of the semi-infinite structure, which is terminated in the $l_{1}l_{2}\ldots l_{n_{2}}$ periodic edge. Obviously, the structure, which will be constructed, cannot be semi-infinite. The only option is to construct a periodic flake representing a ribbon, which can have a large width, and may be terminated with hydrogen atoms so that it can be used for simulations on edges. The idea, therefore, is to find the corresponding 2-2 hexagons represented by their $h_{i}$ vectors pointing to their centers, in accordance with flakes. Then, we can use the same method to find the coordinates of the flake hexagon centers, according to the method described for flakes, and finally, construct the structure.

### 2.4. An Algorithm for the Numbering, Identification, and Generation of Graphene Flakes and Edges

The algorithm we present is based on the algorithm used for the numbering, identification, and generation of graphene pores (see Ref. [34]). Let us assume that we want to find all possible sequences of $l_{1}l_{2}\ldots l_{n_{2}}$, $1\leq l_{i}\leq 4$ representing a flake or the minimum period of an edge composed of $n_{2}$ 2-2 paths. According to what has been reported previously, the total length $L=l_{1}+l_{2}+\ldots +l_{n_{2}}$ of the flake circuit is $L=2n_{2}-6$, while the corresponding length (minimum period) *L* of an edge is $L=2n_{2}$. Thus, as a first step, the algorithm, using a nested DO-loop, searches for all possible combinations of $l_{1}l_{2}\ldots l_{n_{2}}$ for which $l_{1}+l_{2}+\ldots +l_{n_{2}}=2n_{2}-6$ (for flakes), and $l_{1}+l_{2}+\ldots +l_{n_{2}}=2n_{2}$ (for edges). If the algorithm finds such a sequence, it moves to the next step. Otherwise, it rejects that sequence and moves to the next sequence.

Recalling that for flakes $n^{\left(1\right)}\geq 6$, it is obvious that the first digit $l_{1}$ of the minimum image for flakes will always be $l_{1}=1$. Moreover, according to Equation (18) for edges, $n^{\left(1\right)}\geq 0$, with $n^{\left(1\right)}$ being zero only if $n^{\left(3\right)}=n^{\left(4\right)}=0$, i.e., only if $n^{\left(2\right)}\neq 0$. There is only one such case representing the zig-zag edge (i.e., the 222… edge) corresponding to the sole 2-2 path sequence with $l_{1}=2$ and $n_{2}=1$. Thus, excluding that case, the first 2-2 path length of the minimum image will always be $l_{1}=1$. Consequently, with $l_{1}=1$ for both flakes and edges, the search for the possible $l_{1}l_{2}\ldots l_{n_{2}}$ sequences is restricted to the values of the $l_{2}$, $l_{3}$, *…*, $l_{n_{2}}$, reducing the effort by four times. Moreover, recalling Equation (15), one can see that for constant $n^{\left(4\right)}$, if $n^{\left(3\right)}$ increases by 1, $n^{\left(1\right)}$ also increases by 1, and for constant $n^{\left(3\right)}$, if $n^{\left(4\right)}$ increases by 1, $n^{\left(1\right)}$ increases by 2. This means that the sequence $l_{1}l_{2}\ldots l_{n_{2}}$ of a flake is constituted by six paths with length 1, maybe some others with length 2 and/or an amount of (114) and (13) path sections. All these paths or path sections may be mixed with each other in the $l_{1}l_{2}\ldots l_{n_{2}}$ sequence. Thus, any combination of them, which leads to the minimum image of the $l_{1}l_{2}\ldots l_{n_{2}}$ sequence, will start with either $l_{1}=l_{2}=1$ or $l_{1}=1$ and $l_{2}=2$. This means that $l_{2}\neq 3$ or 4; consequently, this reduces the computational effort by another 1/2 (i.e., totally 1/8).

The second step of the algorithm checks whether or not the sequence $l_{1}l_{2}\ldots l_{n_{2}}$ represents the minimum image of the equivalent sequences, representing the same flake or edge. In the serial search for $l_{i}$s, imposed by the nested DO-loops, with the outer one corresponding to $l_{2}$ and the inner ones to $l_{3}$, $l_{4}$, etc., the minimum image will be reached first. Thus, if the algorithm finds a sequence $l_{1}l_{2}\ldots l_{n_{2}}$ not corresponding to the minimum image, it means that the corresponding minimum image has already been found and examined earlier. In that case, the $l_{1}l_{2}\ldots l_{n_{2}}$ sequence is rejected and the algorithm moves to the next $l_{1}l_{2}\ldots l_{n_{2}}$ sequence.

In the third step, the algorithm checks whether or not $l_{1}=l_{2}=l_{3}=l_{4}=1$. As already reported above, such a sequence does not represent a flake or an edge, unless it is the 111111 flake. Therefore, if such a part of the $l_{1}l_{2}\ldots l_{n_{2}}$ sequence is found, the sequence is again rejected, and the algorithm moves to the next $l_{1}l_{2}\ldots l_{n_{2}}$ sequence. It is worth noting that if the first four digits $l_{1}l_{2}l_{3}l_{4}$ of the $l_{1}l_{2}\ldots l_{n_{2}}$ sequence are not all “1”s, then the sub-sequence 1111 will not appear elsewhere in the $l_{1}l_{2}\ldots l_{n_{2}}$ sequence because if such a sub-sequence has to appear, it should appear in the first digits, due to the minimum image convention. Therefore, this check is enough to ensure that a sub-sequence of 1111 will not appear anywhere else in the $l_{1}l_{2}\ldots l_{n_{2}}$ sequence.

In the fourth step, the algorithm calculates the $m_{i,l_{i}}$ values using Equation (14), and in the fifth step, it calculates the $h_{i}$ vectors using Equation (11), i.e., summing the $u_{m_{i,l_{i}}}$ vectors. The calculated $m_{i,l_{i}}$ values and $h_{i}$ vectors are stored for further processing. For convenience, the algorithm starts with $m_{1,1}=0$ and $h_{1}=\left(0,0\right)$, and in the summations of Equation (11), the $u_{m}$ vectors are expressed as $u_{m}=\lambda_{a}a+\lambda_{b}b={\left(\lambda_{a},\lambda_{b}\right)}_{h}$, i.e., $u_{0}=b={\left(0,1\right)}_{h}$, $u_{1}=b-a={\left(-1,1\right)}_{h}$, $u_{2}=-a={\left(-1,0\right)}_{h}$, $u_{3}=-b={\left(0,-1\right)}_{h}$, $u_{4}=a-b={\left(1,-1\right)}_{h}$, and $u_{5}=a={\left(1,0\right)}_{h}$.

In the sixth step, which applies to flakes, but not to edges, the algorithm checks if Equation (13) is satisfied, i.e., if the path is a circuit. If Equation (13) is not satisfied, the sequence $l_{1}l_{2}\ldots l_{n_{2}}$ is rejected, and the algorithm moves to the next sequence. Otherwise, it moves to the seventh and eighth steps, which apply both to flakes and edges, and checks if the circuit or path crosses itself. In the seventh step, the algorithm compares the $h_{i}$ vectors between each other. If ∃i and $i^{\prime }$ for which $h_{i}=h_{i^{\prime }}$, then if (i) $l_{i}\neq 1\neq l_{i^{\prime }}$, or (ii) $l_{i}=l_{i^{\prime }}=1$, but $v_{i,l_{i}}\neq -v_{i^{\prime },l_{i^{\prime }}}$, the sequence $l_{1}l_{2}\ldots l_{n_{2}}$ is rejected, and the algorithm moves to the next sequence. If for all cases for which $h_{i}=h_{i^{\prime }}$, it finds that $l_{i}=l_{i^{\prime }}=1$ and $v_{i,l_{i}}=-v_{i^{\prime },l_{i^{\prime }}}$, the algorithm moves to the next step, finding whether or not two adjacent 2-2 hexagons have a common edge, which belongs to the flake or edge boundary. For that reason, the algorithm checks if ∃i and $i^{\prime }$, for which

- $h_{i^{\prime }}-h_{i}={\left(1,0\right)}_{h}$, and $m_{i,j}=0$, $m_{i^{\prime },j^{\prime }}=3$,
- $h_{i^{\prime }}-h_{i}={\left(0,1\right)}_{h}$, and $m_{i,j}=1$, $m_{i^{\prime },j^{\prime }}=4$,
- $h_{i^{\prime }}-h_{i}={\left(-1,1\right)}_{h}$, and $m_{i,j}=2$, $m_{i^{\prime },j^{\prime }}=5$,
- $h_{i^{\prime }}-h_{i}={\left(-1,0\right)}_{h}$, and $m_{i,j}=3$, $m_{i^{\prime },j^{\prime }}=0$,
- $h_{i^{\prime }}-h_{i}={\left(0,-1\right)}_{h}$, and $m_{i,j}=4$, $m_{i^{\prime },j^{\prime }}=1$,
- $h_{i^{\prime }}-h_{i}={\left(1,-1\right)}_{h}$, and $m_{i,j}=5$, $m_{i^{\prime },j^{\prime }}=2$.

If any of the above cases is true, the 2-2 hexagons determined by $h_{i}$ and $h_{i^{\prime }}$ are adjacent with a common edge, which belongs to the flake boundary. The existence of an $m_{i,j}$ value in a 2-2 path depends on the values of $l_{i}$ and $m_{i,l_{i}}$, according to the relation [34]

(19) $$0\leq mod\left(m_{i,l_{i}}-m_{i,j}+6,6\right)\leq l_{i}-1,$$

which is used by the algorithm. If any of the above cases is true, the $l_{1}l_{2}\ldots l_{n_{2}}$ sequence is rejected, otherwise, the sequence represents a graphene flake, which is numbered and exported as the output. In particular, for edges, the algorithm performs those checks of steps seven and eight, for the minimum period and for two periods, to ensure that the path of the current and the next period do not cross each other. To minimize the computational time for this procedure, the algorithm performs the above tests (i) for the $h_{i}$ and $h_{i^{\prime }}$ vectors, which correspond to the same period, and (ii) for the $h_{i}$ and $h_{i^{\prime }}+L$ vectors, belonging to the current and the next period, respectively, where $L=\sum_{i=1}^{n_{2}+1}u_{i}$ is the period. Therefore, it does not perform those tests for vectors $h_{i}+L$ and $h_{i^{\prime }}+L$, both belonging to the next period, which would not provide any new information that is not known from the tests performed in the current period.

The above procedure is repeated recursively for all possible $l_{1}l_{2}\ldots l_{n_{2}}$ sequences.

If our aim is to find and number the $l_{1}l_{2}\ldots l_{n_{2}}$ sequences representing flakes or edges, the algorithm has to do nothing more than the above steps. If, on the other hand, we also want to find the number of atoms in the flake or the structure (atomic position) of the flake and edge, some extra steps are needed.

To find the number *N* of atoms in a flake, we find the number $n_{h}$ of hexagons in the flake first, using the method described in Section 2.2. Then, using Equation (16), we find *N*. Thus, the eighth step of the algorithm, which will follow each successful $l_{1}l_{2}\ldots l_{n_{2}}$ sequence, is the following: (a) The algorithm finds the maximum $a_{max}$ and the minimum $a_{min}$ coordinates along the a direction of the $h_{i}={\left(\lambda_{a},\lambda_{b}\right)}_{h}$ vectors. If $h^{\prime }={\left(\lambda_{a}^{\prime },\lambda_{b}^{\prime }\right)}_{h}$ is a vector pointing to the center of a hexagon belonging to the flake area, then $a_{min}+1\leq \lambda_{a}^{\prime }\leq a_{max}-1$. (b) For each such $\lambda_{a}^{\prime }$ value, the algorithm shorts the $\lambda_{b}$ coordinates of the $h_{i}=\left(\lambda_{a}^{\prime },\lambda_{b}\right)$ vectors of the 2-2 hexagons in ascending order. (c) Using the value of $m_{i,l_{i}}$, associated with each of those $h_{i}=\left(\lambda_{a}^{\prime },\lambda_{b}\right)$ vectors, the algorithm finds if for that for *i*, $\exists \;j:\;m_{i,j}=1$ or 4. The cases for which for a certain *i*,

- ∃j and $j^{\prime }\;:\;m_{i,j}=1$ and $m_{i,j^{\prime }}=4$, are those for which $l_{i}=4$ and $m_{i,l_{i}}=1$ or 4,
- $\exists \;j:\;m_{i,j}=1$, but $∄\;j:\;m_{i,j}=4$ are the following:
  - $l_{i}=4$ and $m_{i,l_{i}}=2$ or 3,
  - $l_{i}=3$ and $m_{i,l_{i}}=1$, 2 or 3,
  - $l_{i}=2$ and $m_{i,l_{i}}=1$ or 2, and
  - $l_{i}=1$ and $m_{i,l_{i}}=1$.
- $\exists \;j:\;m_{i,j}=4$, but $∄\;j:\;m_{i,j}=1$ are the following:
  - $l_{i}=4$ and $m_{i,l_{i}}=5$ or 0,
  - $l_{i}=3$ and $m_{i,l_{i}}=4$, 5 or 0,
  - $l_{i}=2$ and $m_{i,l_{i}}=4$ or 5, and
  - $l_{i}=1$ and $m_{i,l_{i}}=4$.

The details of these results can be found in Ref. [34]. The values of $m_{i,l_{i}}$ are calculated in the fourth step of the algorithm, see above. Having this information, and the ordered $\lambda_{b}$ values, the algorithm finds the number of hexagons for each $\lambda_{a}^{\prime }$ value in the range $\left[a_{min}+1,a_{max}-1\right]$ and sums them to find $n_{h}$. During this step, the algorithm stores the coordinates ${\left(\lambda_{a}^{\prime },\lambda_{b}^{\prime }\right)}_{h}$ of the $h^{\prime }$ vectors, pointing to the center of the flake hexagons. Those vectors are used in the next step for the determination of the flake structure.

Considering that the hexagonal graphene structure can be constructed by vectors $h_{\lambda_{a},\lambda_{b}}+v_{4}$ and $h_{\lambda_{a},\lambda_{b}}+v_{5}$, where $h_{\lambda_{a},\lambda_{b}}={\left(\lambda_{a},\lambda_{b}\right)}_{h}=\lambda_{a}a+\lambda_{b}b$, $\forall \;\lambda_{a},\lambda_{b}\in Z$, the flake structure can be found using the $h^{\prime }={\left(\lambda_{a}^{\prime },\lambda_{b}^{\prime }\right)}_{h}$ vectors, which were found in the previous step, and the vectors $h_{i}$ corresponding to the 2-2 hexagons. Thus, the positions of the flake atoms are determined by (i) the vectors $R_{1}^{\prime }=h^{\prime }+v_{4}$ and $R_{2}^{\prime }=h^{\prime }+v_{5}$, for each $h^{\prime }$ vector, (ii) the vectors $R_{1}=h_{i}+v_{4}$, if $\exists \;j\;:\;m_{i,j}=4$, for $1\leq j\leq l_{i}$, and (iii) the vectors $R_{2}=h_{i}+v_{5}$ if $\exists \;j\;:\;m_{i,j}=5$, for $1\leq j\leq l_{i}$. In a similar way, we may find the structure of a ribbon that has the edge of interest, as described above.

## 3. Results and Discussion

Using the algorithm described above, we developed two Fortran codes, which can be found at https://github.com/fthenak/Graphene-Pores-Flakes-Edges (accessed on 3 July 2023). The first one with the name “*pore_flake_edge_generation.f90*” takes as input (i) the system kind (i.e., pore, flake, or edge) and (ii) the number $n_{2}$ of 2-FCAs in the boundary of each system, and provides as output (i) the number $n_{2}\;\;:\;\;m$ of each pore, flake, or edge, (ii) the corresponding minimum image of the $l_{1}l_{2}\ldots l_{n_{2}}$ sequence, and (iii) the length *L* of the pore or flake circuit, or periodic edge path. Moreover, for flakes, it provides (iv) the number of hexagons of the flake and (v) the number of atoms constituting the flake, and for pores, (iv) the number of hexagons in the vacuum area and (v) the number of vacancies. The second code with the name “*pore_flake_edge_structure.f90*” takes as input (i) the system kind (i.e., pore, flake, or edge), (ii) the number $n_{2}$ of 2-FCAs in the boundary of each system, and (iii) the sequence $l_{1}l_{2}\ldots l_{n_{2}}$ in separated digits. For the case of pores, a width *d* of atoms around the pore is also an input, considering that pores are periodically arranged in the infinite graphene sheet. If that sequence corresponds to the selected system (pore, flake, or edge), it provides as output (i) the structure in the xyz format, which is stored in a file under the name “*structure.xyz*”, (ii) the corresponding minimum image of the sequence, (iii) and the length *L* of the circuit or path. Moreover, (a) for a flake, it provides (iv) the number of hexagons and (v) the number of atoms in the flake, (b) for a pore, (iv) the number of hexagons in the vacuum area and (v) the number of vacancies, and (c) for an edge, (iv) the number of ribbon atoms and the vector period L. Those codes are extensions of the codes pore_generation.f90 and pore_structure.f90, which were developed exclusively for pores and can be found in the Supplementary Information of Ref. [34].

Using the first code, we find the number of graphene flakes ($N_{f}$) and periodic edges ($N_{e}$) for different numbers $n_{2}$ of 2-FCAs in their boundaries. In Table 1, we present those numbers as functions of $n_{2}$. For comparison and completeness, in the same table, we also present the corresponding numbers of different graphene pores ($N_{p}$), which are presented in Ref. [34]. In the same table, in parenthesis, we present the number of pores ($d_{p}$) and flakes ($d_{f}$), which have a dual.

Recalling that the length *L* of the circuit of a flake or a pore is the same as the length of its dual, and for a pore $L=2n_{2p}+6$, while for a flake $L=2n_{2f}-6$, we conclude that for flakes and pores with the same length *L*, $n_{2f}=n_{2p}+6$. This means that the number of flakes and pores with the same length *L* should have the same number of duals. This is clearly shown in Table 1, where the number of pores that have a dual for $n_{2}=n_{2p}$ is the same as the number of flakes, which have a dual for $n_{2}=n_{2f}=n_{2p}+6$, e.g., for $n_{2}=12$, $d_{p}=517$, and for $n_{2}=18=12+6$, $d_{f}=517$. Moreover, Table 1 shows that most of the pores have a dual, contrary to flakes, where only a few of them have a dual, indicating that for the same *L*, $N_{f}>>N_{p}$, while for the same $n_{2}$, $N_{p}>>N_{f}$.

**Figure 5:**
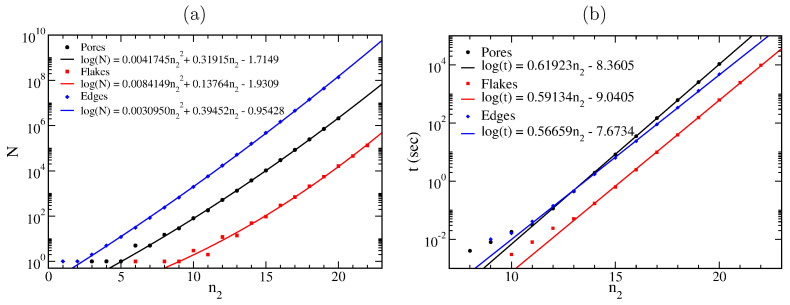
(**a**) Number *N* of pores, flakes, and periodic edges as functions of the number $n_{2}$ of 2-fold coordinated atoms at the pore, flake, and edge boundaries, respectively. (**b**) Computational time needed to find the number *N* of pores, flakes, and edges having $n_{2}$ 2-fold coordinated atoms in their boundaries.

Using a semi-logarithmic plot, we present the results of Table 1 in Figure 5a, and we fit logarithmic quadratic functions of the form $\log\left(N\right)=an_{2}^{2}+bn_{2}+c$ to those points. The fitted functions are presented in the legends of that figure. As one can see, they fit almost perfectly (particularly for large $n_{2}$ values) to the $\left(N,n_{2}\right)$ points. It is worth noting that a simple logarithmic linear function of the form $\log\left(N\right)=an_{2}+b$ does not have the same fitting quality as the logarithmic quadratic one, although the coefficients of the quadratic terms in all three cases are relatively small.

In Table 2 and Table 3, we present the minimum image sequences $l_{1}l_{2}\ldots l_{n_{2}}$ of flakes with $n_{2}\leq 15$ and periodic edges with $n_{2}\leq 7$, respectively, which were found using the above-mentioned *pore_flake_edge_generation.f90* code. In the same tables, the corresponding flake and edge numbers $n_{2}\;\;:\;\;m$ are also presented, as well as the length *L* of the circuit of the flakes and paths of the edge periods, respectively. Moreover, Table 2 also contains the number $n_{h}$ of flake hexagons and the number *N* of atoms in the flake. The star symbol (*), which appears in some lines of Table 2, indicates that the flake corresponding to that line has a dual.

For completeness, we present in Figure 5b the computational time versus $n_{2}$ for the execution of the *pore_flake_edge_generation.f90* code, for the generation and numbering of the pore, flake, and edge $l_{1}l_{2}\ldots l_{n_{2}}$ sequences, using an Intel(R) Xeon(R) Gold 6132 CPU @ 2.60 GHz processor, with the ability of 761.6 GFlops. As one can see, the computational time for those calculations rises exponentially with $n_{2}$, as expected. The computational time used for flakes seems to be ≈3 times smaller than that for pores and edges, which can be explained due to the reduction of searches for the $l_{1}l_{2}\ldots l_{n_{2}}$ sequences, only for $l_{3},l_{4},\ldots ,l_{n_{2}}$, since $l_{1}=1$ and $l_{2}$ is either 1 or 2, while pore $l_{1}=1$ or 2, and edge $l_{1}=1$. Moreover, for the edges, the algorithm does not have to check if the path is closed or not, contrary to the flakes and pores, while for flakes and pores, it also finds the number of flake atoms and the number of vacancies, respectively, which explains the lower slope of the fitted logarithmic lines for edges compared to the slopes for flakes and pores.

## 4. Conclusions

Generalizing our nomenclature scheme for graphene pores, we propose a general, unique, systematic, accurate, and simple nomenclature scheme for naming graphene flakes and edges, which is based on the arrangement of 2-fold coordinated atoms in their boundaries. In the proposed nomenclature scheme, flakes and edges are named after the minimum images of sequences $l_{1}l_{2}\ldots l_{n_{2}}$ of “lengths" $l_{i}$ of the paths along the boundary connecting 2-fold coordinated atoms, in accordance with the nomenclature scheme for pores, where the term “length" is used from the point of view of graph theory. We also systematically study the geometric features of graphene flakes and edges utilizing ideas from graph theory. Using the results of that study, we present an algorithm for their generation, numbering, and identification, accompanied by Fortran codes implementing that algorithm. Using these codes, we found (i) the number of pores and edges for $n_{2}$ up to 20, and the number of flakes for $n_{2}$ up to 22, and (ii) the $l_{1}l_{2}\ldots l_{n_{2}}$ sequences of all possible flakes with $n_{2}\leq 15$ and edges with $n_{2}\leq 7$.

The proposed nomenclature scheme can be easily expanded to name pores, flakes, and edges of other two-dimensional systems (e.g., the planar honeycomb structure of BN), as well as functionalized pores, flakes, and edges, according to the scheme described in our previous work, thus opening up a new window in the nomenclature of such systems.

## Figures and Tables

**Table 1:** Number of pores ($N_{p}$), flakes ($N_{f}$), and periodic edges ($N_{e}$) for $n_{2}$ 2-fold coordinated atoms in their boundary. The columns under $d_{p}$ and $d_{f}$ (the numbers in parenthesis) are the numbers of pores and flakes, respectively, which have a dual.

$n_{2}$	$N_{p}$	$d_{p}$	$N_{f}$	$d_{f}$	$N_{e}$
1	-	-	-	-	1
2	-	-	-	-	1
3	1	(1)	-	-	2
4	1	(1)	-	-	5
5	1	(1)	-	-	12
6	5	(5)	1	(0)	31
7	5	(5)	-	-	84
8	15	(15)	1	(0)	239
9	29	(29)	1	(1)	666
10	81	(81)	3	(1)	1962
11	181	(181)	2	(1)	5746
12	523	(517)	12	(5)	17,253
13	1327	(1305)	14	(5)	51,928
14	3790	(3672)	49	(15)	158,154
15	10,408	(9944)	95	(29)	483,238
16	29,882	(28,028)	298	(81)	1,486,402
17	84,932	(78,180)	701	(181)	4,587,114
18	246,507	(222,195)	2099	(517)	14,215,986
19	714,738	(630,379)	5546	(1305)	44,184,779
20	2,094,636	(1,805,397)	16,279	(3672)	137,748,147
21			45,583	(9994)	
22			133,454	(28,028)	

**Table 2:** All possible flakes for $n_{2}\leq 15$. The table shows the flake numbering $n_{2}\;\;:\;\;m$, the sequences $l_{1}l_{2}\ldots l_{n_{2}}$, the number of atoms *N* and hexagons $n_{h}$ in the flake, and the length *L* of the flake circuit. The star symbol (*), which appears in some lines, indicates that that flake has a dual.

$n_{2}\;:\;m$	Sequence	*N*	$n_{h}$	*L*	$n_{2}\;:\;m$	Sequence	*N*	$n_{h}$	*L*	$n_{2}\;:\;m$	Sequence	*N*	$n_{h}$	*L*
06:01	111111	6	1	6	14:27	11123112121213	26	7	22	15:38	111222121131123	29	8	24
08:01	11121112	10	2	10	14:28	11131122121123	26	7	22	15:39	111222121211232	27	7	24
09:01 *	112112112	13	3	12	14:29	11131131113113	24	6	22	15:40	111222121211313	29	8	24
10:01	1112121113	14	3	14	14:30	11131131121213	26	7	22	15:41	111222121212123	31	9	24
10:02	1112211122	14	3	14	14:31	11131211311213	26	7	22	15:42	111222211123113	25	6	24
10:03 *	1121211212	16	4	14	14:32	11131212121213	28	8	22	15:43	111222211131213	27	7	24
11:01	11122112113	17	4	16	14:33	11211222112114	24	6	22	15:44	111222211211322	25	6	24
11:02 *	11212121122	19	5	16	14:34	11211231121123	24	6	22	15:45	111222211212132	27	7	24
12:01	111212121114	18	4	18	14:35 *	11211312121123	26	7	22	15:46	111222211212213	29	8	24
12:02	111212211123	18	4	18	14:36 *	11212122121123	28	8	22	15:47	111231112311123	25	6	24
12:03	111213111213	18	4	18	14:37 *	11212131121213	28	8	22	15:48	111231113121123	27	7	24
12:04	111221211213	20	5	18	14:38 *	11212211311213	28	8	22	15:49	111231121221123	29	8	24
12:05	111222111222	18	4	18	14:39 *	11212212113113	28	8	22	15:50	111231122112132	27	7	24
12:06	111311131113	18	4	18	14:40 *	11212212121213	30	9	22	15:51	111231122112213	29	8	24
12:07	111311212113	20	5	18	14:41 *	11212221121222	28	8	22	15:52	111231211213113	27	7	24
12:08 *	112113112113	20	5	18	14:42 *	11213112211222	28	8	22	15:53	111231211221213	29	8	24
12:09 *	112121212113	22	6	18	14:43 *	11213121121312	26	7	22	15:54	111311132112114	25	6	24
12:10 *	112122112122	22	6	18	14:44 *	11213121122122	28	8	22	15:55	111311213112114	27	7	24
12:11 *	112211221122	22	6	18	14:45 *	11221131122113	28	8	22	15:56	111311221131114	27	7	24
12:12 *	121212121212	24	7	18	14:46 *	11221212122113	30	9	22	15:57	111311221212114	29	8	24
13:01	1112122112114	21	5	20	14:47 *	11221212211222	30	9	22	15:58	111311222112123	29	8	24
13:02	1112131121123	21	5	20	14:48 *	11221221122122	30	9	22	15:59	111311311221123	29	8	24
13:03	1112211221114	21	5	20	14:49 *	12121221212122	32	10	22	15:60	111311312112213	29	8	24
13:04	1112211311123	21	5	20	15:01	111212131121124	25	6	24	15:61	111312113121123	29	8	24
13:05	1112212121123	23	6	20	15:02	111212211311124	25	6	24	15:62	111312121221123	31	9	24
13:06	1112221113113	21	5	20	15:03	111212212121124	27	7	24	15:63	111312122112213	31	9	24
13:07	1112221121132	21	5	20	15:04	111212221113114	25	6	24	15:64	112112221211214	27	7	24
13:08	1112221121213	23	6	20	15:05	111212221121133	25	6	24	15:65	112112311212114	27	7	24
13:09	1113112211213	23	6	20	15:06	111212221121214	27	7	24	15:66	112112312112123	27	7	24
13:10 *	1121131211213	23	6	20	15:07	111212311122114	25	6	24	15:67 *	112113113112114	27	7	24
13:11 *	1121212211213	25	7	20	15:08	111212311211223	25	6	24	15:68 *	112113121212114	29	8	24
13:12 *	1121221122113	25	7	20	15:09	111213112211133	25	6	24	15:69 *	112113122112123	29	8	24
13:13 *	1121221211222	25	7	20	15:10	111213112211214	27	7	24	15:70 *	112113211221123	29	8	24
13:14 *	1122121212122	27	8	20	15:11	111213113111223	25	6	24	15:71 *	112121221212114	31	9	24
14:01	11121212211124	22	5	22	15:12	111213121122114	27	7	24	15:72 *	112121222112123	31	9	24
14:02	11121213111214	22	5	22	15:13	111213121211223	27	7	24	15:73 *	112121311221123	31	9	24
14:03	11121221211133	22	5	22	15:14	111213211131123	25	6	24	15:74 *	112121312112132	29	8	24
14:04	11121221211214	24	6	22	15:15	111213211211313	25	6	24	15:75 *	112121312112213	31	9	24
14:05	11121222111223	22	5	22	15:16	111213211212123	27	7	24	15:76 *	112122113121123	31	9	24
14:06	11121231112123	22	5	22	15:17	111214111221123	25	6	24	15:77 *	112122121221123	33	10	24
14:07	11121311131114	22	5	22	15:18	111214112112213	25	6	24	15:78 *	112122122112132	31	9	24
14:08	11121311212114	24	6	22	15:19	111221122211124	25	6	24	15:79 *	112122122112213	33	10	24
14:09	11121312111313	22	5	22	15:20	111221131211214	27	7	24	15:80 *	112122211213113	31	9	24
14:10	11121312112123	24	6	22	15:21	111221132111223	25	6	24	15:81 *	112122211221132	31	9	24
14:11	11121321112213	22	5	22	15:22	111221212211133	27	7	24	15:82 *	112122211221213	33	10	24
14:12	11122113112114	24	6	22	15:23	111221212211214	29	8	24	15:83 *	112122212112222	31	9	24
14:13	11122121131114	24	6	22	15:24	111221213111223	27	7	24	15:84 *	112131121311213	31	9	24
14:14	11122121212114	26	7	22	15:25	111221221121142	25	6	24	15:85 *	112131122113113	31	9	24
14:15	11122122111232	22	5	22	15:26	111221221122114	29	8	24	15:86 *	112131122121213	33	10	24
14:16	11122122111313	24	6	22	15:27	111221221211223	29	8	24	15:87 *	112131211222113	31	9	24
14:17	11122122112123	26	7	22	15:28	111221311131123	27	7	24	15:88 *	112131212121222	33	10	24
14:18	11122131112213	24	6	22	15:29	111221311211232	25	6	24	15:89 *	112211312121213	33	10	24
14:19	11122211221123	26	7	22	15:30	111221311211313	27	7	24	15:90 *	112212122113113	33	10	24
14:20	11122212112132	24	6	22	15:31	111221311212123	29	8	24	15:91 *	112212122121213	35	11	24
14:21	11122212112213	26	7	22	15:32	111222111321114	25	6	24	15:92 *	112212211222113	33	10	24
14:22	11122221112222	22	5	22	15:33	111222112131114	27	7	24	15:93 *	112212212121222	35	11	24
14:23	11123111311132	22	5	22	15:34	111222112212114	29	8	24	15:94 *	112221122211222	33	10	24
14:24	11123111311213	24	6	22	15:35	111222113111232	25	6	24	15:95 *	121221212212122	37	12	24
14:25	11123112113113	24	6	22	15:36	111222113111313	27	7	24					
14:26	11123112121132	24	6	22	15:37	111222113112123	29	8	24					

**Table 3:** All possible periodic edges with a period including $n_{2}\leq 7$ 2-fold coordinated atoms at the edge boundary. The table shows the number $n_{2}\;\;:\;\;m$ of each edge, the sequence $l_{1}l_{2}\ldots l_{n_{2}}$ corresponding to the minimum period, and the corresponding length *L* of the periodic edge path, ($L=2n_{2}$).

$n_{2}\;:\;m$	Sequence	*L*	$n_{2}\;:\;m$	Sequence	*L*	$n_{2}\;:\;m$	Sequence	*L*	$n_{2}\;:\;m$	Sequence	*L*	$n_{2}\;:\;m$	Sequence	*L*
01:01	2	2	06:08	112224	12	07:05	1112333	14	07:33	1122422	14	07:61	1212323	14
02:01	13	4	06:09	112233	12	07:06	1112342	14	07:34	1123124	14	07:62	1212332	14
03:01	114	6	06:10	112242	12	07:07	1112414	14	07:35	1123133	14	07:63	1212413	14
03:02	123	6	06:11	112314	12	07:08	1112423	14	07:36	1123142	14	07:64	1213124	14
04:01	1124	8	06:12	112323	12	07:09	1113134	14	07:37	1123214	14	07:65	1213133	14
04:02	1133	8	06:13	112332	12	07:10	1113143	14	07:38	1123223	14	07:66	1213214	14
04:03	1214	8	06:14	112413	12	07:11	1113224	14	07:39	1123232	14	07:67	1213223	14
04:04	1223	8	06:15	113124	12	07:12	1113233	14	07:40	1123313	14	07:68	1221224	14
04:05	1232	8	06:16	113133	12	07:13	1113314	14	07:41	1124114	14	07:69	1221233	14
05:01	11134	10	06:17	113214	12	07:14	1114124	14	07:42	1124123	14	07:70	1221242	14
05:02	11224	10	06:18	113223	12	07:15	1121144	14	07:43	1124213	14	07:71	1221314	14
05:03	11233	10	06:19	121224	12	07:16	1121234	14	07:44	1131134	14	07:72	1221323	14
05:04	11242	10	06:20	121233	12	07:17	1121243	14	07:45	1131224	14	07:73	1222124	14
05:05	11314	10	06:21	121242	12	07:18	1121324	14	07:46	1131233	14	07:74	1222133	14
05:06	11323	10	06:22	121314	12	07:19	1121333	14	07:47	1131314	14	07:75	1222214	14
05:07	12124	10	06:23	121323	12	07:20	1121342	14	07:48	1131323	14	07:76	1222223	14
05:08	12133	10	06:24	122133	12	07:21	1121414	14	07:49	1131413	14	07:77	1222232	14
05:09	12214	10	06:25	122214	12	07:22	1121423	14	07:50	1132124	14	07:78	1222313	14
05:10	12223	10	06:26	122223	12	07:23	1121432	14	07:51	1132133	14	07:79	1222322	14
05:11	12232	10	06:27	122232	12	07:24	1122134	14	07:52	1132214	14	07:80	1223123	14
05:12	12313	10	06:28	122313	12	07:25	1122143	14	07:53	1132223	14	07:81	1223132	14
06:01	111234	12	06:29	122322	12	07:26	1122224	14	07:54	1133114	14	07:82	1223213	14
06:02	111243	12	06:30	123132	12	07:27	1122233	14	07:55	1141214	14	07:83	1231232	14
06:03	111324	12	06:31	123213	12	07:28	1122242	14	07:56	1212134	14	07:84	1231313	14
06:04	111333	12	07:01	1112144	14	07:29	1122314	14	07:57	1212224	14			
06:05	111414	12	07:02	1112234	14	07:30	1122323	14	07:58	1212233	14			
06:06	112134	12	07:03	1112243	14	07:31	1122332	14	07:59	1212242	14			
06:07	112143	12	07:04	1112324	14	07:32	1122413	14	07:60	1212314	14			

## Data Availability

The data that support the findings of this study (i.e., the two Fortran codes described in Section 3) are openly available on github.com at https://github.com/fthenak/Graphene-Pores-Flakes-Edges (accessed on 3 July 2023).

## Abbreviations

The following abbreviations are used in this manuscript:

2-FCA	two-fold coordinated atom
3-FCA	three-fold coordinated atom
